# Supplementary figures and images for: Comparative analysis of WC1.1+ and WC1.2+ γδ T cell subset responses from cattle naturally infected with Mycobacterium bovis to repeat stimulation with mycobacterial antigens
Source: PLoS One. 2024 Dec 12;19(12):e0311854. doi: 10.1371/journal.pone.0311854 (PMC11637235; doi:10.1371/journal.pone.0311854)

bTB + Stimulated WC1.1<sup>+</sup> γδ T Cell vs Stimulated WC1.2<sup>+</sup> γδ T Cell

EnhancedVolcano

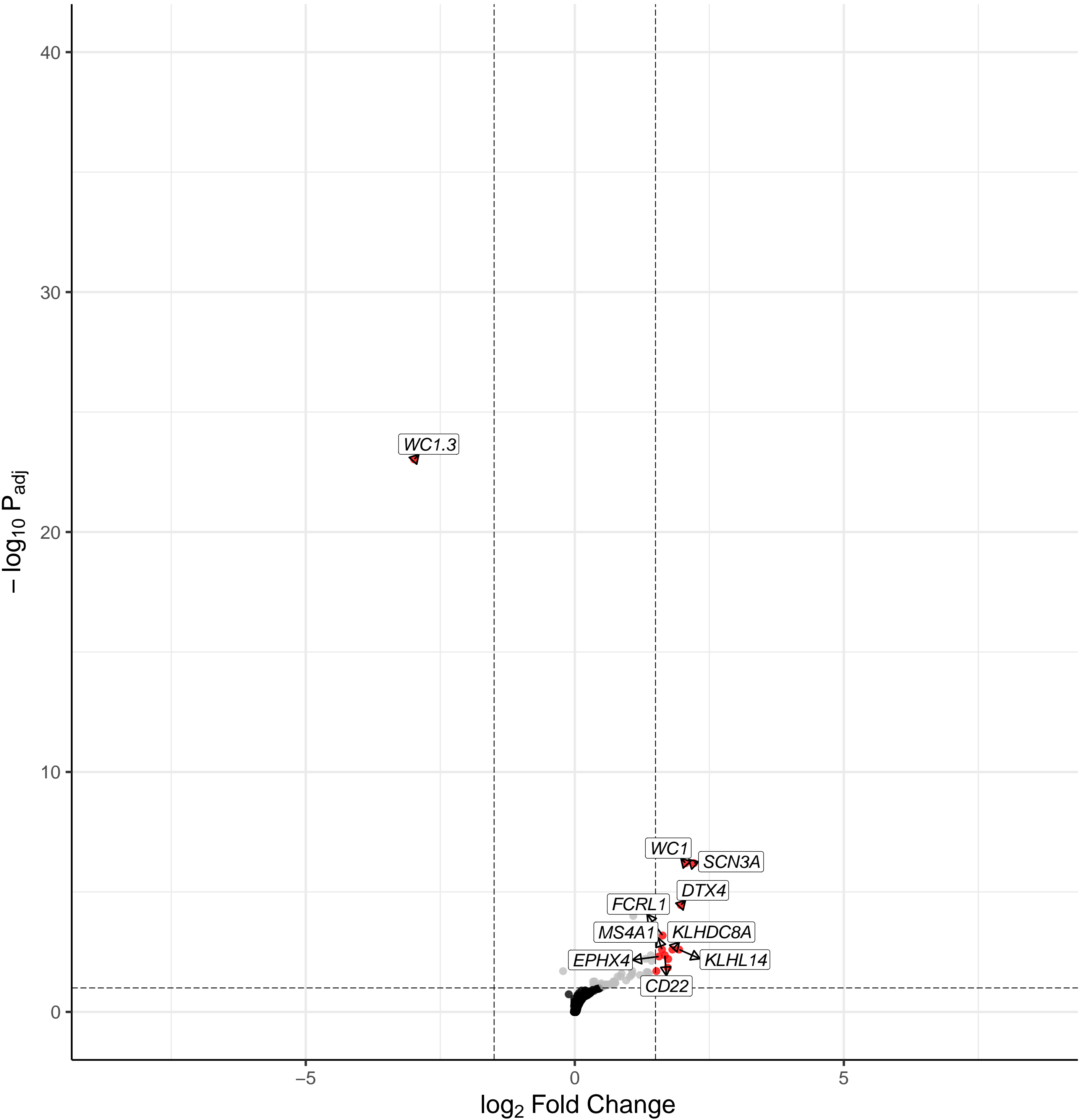

Supplement: S1 Fig — Red dots represent both upregulated and downregulated genes with |log2FC| ≥ 1.5 and FDR-Padj. < 0.10 (above the black dashed lines). Grey dots indicate DEGs with FDR-Padj. < 0.10 but |log2FC| < 1.5. Black dots represent non-significant genes below the threshold of |log2FC| ≥ 1.5 and FDR-Padj. < 0.10. (PDF) [file pone.0311854.s001.pdf]
