# Supplementary material for: Comparative analysis of WC1.1+ and WC1.2+ γδ T cell subset responses from cattle naturally infected with Mycobacterium bovis to repeat stimulation with mycobacterial antigens
Source: PLoS One. 2024 Dec 12;19(12):e0311854. doi: 10.1371/journal.pone.0311854 (PMC11637235; doi:10.1371/journal.pone.0311854)
Supplement: Attachment — Submitted filename: Response to reviewers.docx [file pone.0311854.s006.docx]

PLOS ONE Decision: Revision required [PONE-D-24-33992] - [EMID:9ac9cf402283514b]

09-09-24

Dear Dr Chang,

We very much appreciate the positive responses and detailed analysis provided on our submission by yourself and reviewers. We have addressed all the points raised in the revised version (attached) and below is a point-by-point response. The manuscript is now much improved, and we hope that the revised version will be considered for publication in *PLOS ONE.*

*Regards,*

*Dr Kieran Meade*

**Point-by-point response**

**Reviewer Comments:**

**Reviewer 1**

*Reviewer 1*

The work “Comparative analysis of WC1.1+ and WC1.2+ γδ T cell subset responses from cattle naturally infected with Mycobacterium bovis to repeat stimulation with mycobacterial antigens” report some interesting findings in specific T cell populations from natural tb-infected cows.
Although I think the manuscript is well written and straightforward, I found that the lack of line numbers makes it difficult to point out errors and revision requests.
Here are some comments to be considered by the authors before publication.

**Author response:** We thank the reviewer for their positive comments on our manuscript. We have now added line numbers to make the review process easier.

General comments on manuscript:
- The use of bulk RNAseq strategy to characterize γδ T cells response to PPD is still a useful analysis method to explore coding transcriptome, and interesting data can therefore be collected by this approach. However, considering currently available single-cell RNAseq technologies, I suggest the author to revise the conclusion sections of their manuscript and to add some comments in this regard.

**Author response:** We acknowledge that single cell sequencing is very much becoming the preferred technology for interrogating the transcriptome, particularly in human and model organism studies. However, financial constraints in our livestock studies meant that we started with a bulk RNA-sequencing approach and circumvented some of the issues we faced previously with cellular heterogeneity by focusing on a single cell sub-type. Now that we have uncovered significant differences, we do plan to use SC-seq for future research on these cells. In light of the reviewers comments we now state ““*and further confirmatory research in additional samples and potentially incorporating the use of single cell sequencing is needed to more fully elucidate the specific functions of these critically important γδ T cells under various disease conditions in livestock*.” in the discussion section of the revised manuscript.

- Moreover, in order to further enrich the discussion of their paper, I invite the authors also to report and cite some studies regarding non-coding transcriptome (lncRNA.miRNA,etc..) that seems to modulate specific gene expression pathways related to M. bovis infection.

**Author response:** In light of the reviewers’ comments we now include reference to studies on the non-coding transcriptome although these studies are limited in the cell type and species under investigation. We now state “*The γδ T cell subpopulation has attracted significant research attention across species due to their developmental and functional plasticity. Although transcriptomics approaches have been used to interrogate the effects of both coding and non-coding transcript changes [66, 67] on cell function, a lot remains to be discovered [9, 68], particularly in livestock and non-model organisms*.” in the discussion section of the revised manuscript.

- How the fixed time course of the PPD exposure (6 hours) was chosen by the authors? Did the authors consider also other PPD incubation times?

**Author response:** The 6-hour time point was chosen in order to detect early transcriptomic responses that may be missed during a longer incubation. In addition, recent studies have used 4 hours (1) showing early activation of these γδ T cells in cattle.

- Regarding recorded data, I think a confirmatory analysis by qPCR (Real Time PCR, digital PCR) on most discriminant genes for both WC1.1+ and WC1.2+ is still useful and should be considered for validation of DEG found by sequencing analysis. This aspect is crucial also in order to extend in the future their findings to supplementary field samples without the use of costly RNAseq. Why the author did not include a confirmatory gene expression study in their study? I invite them to justify their choices in the result/discussion sections.

**Author response:** We thank the reviewer for this recommendation, and functional validation will form an important part of the pathway specific analyses that we plan for future work. We believe that RT-qPCR is unnecessary for validation of results from the same set of biological samples because RNA-seq has been long understood to be highly reproducible with relatively little technical variation (2). Multiple previous studies have assessed the concordance between RNA-seq and RT-qPCR and the added advantage is that RNA-seq is recommended to permit consistent comparisons across laboratories and platforms (3). Validation of RNA-seq results with RT-qPCR would be informative and appropriate if an independent set of biological replicates is available, which are not available at this time. A recent editorial on the issue concluded that (4) a requirement for validation of genome-scale expression studies using RNA-seq stems from the legacy of the much less reproducible gene expression microarrays, particularly the primitive spotted arrays that were widely used 20 years ago. Modern RNA-seq laboratory workflows and analytical methods are significantly more robust than these older methods and the same set of samples do not require validation by RT-qPCR. This large-scale (20 individual RNA-seq libraries) transcriptomic analysis was used to identify specific targets for which detailed functional validation and that work is now on-going.

In light of the reviewers comments we have added the following statement to the conclusion of the revised manuscript “*and further confirmatory research in additional samples and potentially incorporating the use of single cell sequencing is needed to more fully elucidate the specific functions of these critically important γδ T cells under various disease conditions in livestock*.”

Specific comments to manuscript sections:
- Please check through the text the use of italics for scientific names (for example B. taurus in the section RNA-seq Data Processing and Analysis).
- In the section GO and KEGG Enrichment Analyses please change this sentence “Lists of genes with increased |log2FC| ≥ 1.5 and FDR-Padj. < 0.10) or decreased (|log2FC| ≥ 1.5, and FDR-Padj. < 0.10)..” with “Lists of genes with increased or decreased expression (|log2FC| ≥ 1.5 andFDR-Padj. < 0.10)…”

**Author response:** We have made the corrections as suggested by reviewer 1 in the revised manuscript.

**References:**

1.	Samuel BER, Diaz FE, Maina TW, Corbett RJ, Tuggle CK, McGill JL. Evidence of innate training in bovine gammadelta T cells following subcutaneous BCG administration. Front Immunol. 2024;15:1423843.

2.	Marioni JC, Mason CE, Mane SM, Stephens M, Gilad Y. RNA-seq: an assessment of technical reproducibility and comparison with gene expression arrays. Genome Res. 2008;18(9):1509-17.

3.	Xu J, Gong B, Wu L, Thakkar S, Hong H, Tong W. Comprehensive Assessments of RNA-seq by the SEQC Consortium: FDA-Led Efforts Advance Precision Medicine. Pharmaceutics. 2016;8(1).

4.	Coenye T. Do results obtained with RNA-sequencing require independent verification? Biofilm. 2021;3:100043.
